# Development of Methods for Cross-Sectional HIV Incidence Estimation in a Large, Community Randomized Trial

**DOI:** 10.1371/journal.pone.0078818

**Published:** 2013-11-13

**Authors:** Oliver Laeyendecker, Michal Kulich, Deborah Donnell, Arnošt Komárek, Marek Omelka, Caroline E. Mullis, Greg Szekeres, Estelle Piwowar-Manning, Agnes Fiamma, Ronald H. Gray, Tom Lutalo, Charles S. Morrison, Robert A. Salata, Tsungai Chipato, Connie Celum, Erin M. Kahle, Taha E. Taha, Newton I. Kumwenda, Quarraisha Abdool Karim, Vivek Naranbhai, Jairam R. Lingappa, Michael D. Sweat, Thomas Coates, Susan H. Eshleman

**Affiliations:** 1 National Institute of Allergy and Infectious Diseases, National Institutes of Health, Bethesda, Maryland, United States of America; 2 Department of Medicine, Johns Hopkins University School of Medicine, Baltimore, Maryland, United States of America; 3 Department of Probability and Statistics, Faculty of Mathematics and Physics, Charles University in Prague, Prague, Czech Republic; 4 Statistical Center for HIV/AIDS Research and Prevention, Fred Hutchinson Cancer Research Center, Seattle, Washington, United States of America; 5 UCLA Program in Global Health, University of California Los Angeles, Los Angeles, California, United States of America; 6 Department of Pathology, Johns Hopkins University, School of Medicine, Baltimore, Maryland, United States of America; 7 Department of Epidemiology, Johns Hopkins Bloomberg School of Public Health, Baltimore, Maryland, United States of America; 8 Rakai Health Science Program, Entebbe, Uganda; 9 Clinical Sciences, Family Health International, Durham, North Carolina, United States of America; 10 Division of Infectious Diseases and HIV Medicine, Case Western Reserve University, Cleveland, Ohio, United States of America; 11 University Hospitals, Case Medical Center, Cleveland, Ohio, United States of America; 12 University of Zimbabwe, Harare, Zimbabwe; 13 Department of Global Health, Medicine and Epidemiology, University of Washington, Seattle, Washington, United States of America; 14 Department of Epidemiology, University of Washington, Seattle, Washington, United States of America; 15 Department of Epidemiology, Mailman School of Public Health, Columbia University, New York, New York, United States of America; 16 Centre for the AIDS Programme of Research In South Africa (CAPRISA), Doris Duke Medical Research Institute, Nelson R Mandela School of Medicine, University of KwaZulu-Natal, Congella, South Africa; 17 Departments of Global Health, Medicine and Pediatrics, University of Washington, Seattle, Washington, United States of America; 18 Department of Psychiatry and Behavioral Sciences, the Medical University of South Carolina, Charleston, South Carolina, United States of America; University of Athens, Medical School, Greece

## Abstract

**Background:**

Accurate methods of HIV incidence determination are critically needed to monitor the epidemic and determine the population level impact of prevention trials. One such trial, Project Accept, a Phase III, community-randomized trial, evaluated the impact of enhanced, community-based voluntary counseling and testing on population-level HIV incidence. The primary endpoint of the trial was based on a single, cross-sectional, post-intervention HIV incidence assessment.

**Methods and Findings:**

Test performance of HIV incidence determination was evaluated for 403 multi-assay algorithms [MAAs] that included the BED capture immunoassay [BED-CEIA] alone, an avidity assay alone, and combinations of these assays at different cutoff values with and without CD4 and viral load testing on samples from seven African cohorts (5,325 samples from 3,436 individuals with known duration of HIV infection [1 month to >10 years]). The mean window period (average time individuals appear positive for a given algorithm) and performance in estimating an incidence estimate (in terms of bias and variance) of these MAAs were evaluated in three simulated epidemic scenarios (stable, emerging and waning). The power of different test methods to detect a 35% reduction in incidence in the matched communities of Project Accept was also assessed. A MAA was identified that included BED-CEIA, the avidity assay, CD4 cell count, and viral load that had a window period of 259 days, accurately estimated HIV incidence in all three epidemic settings and provided sufficient power to detect an intervention effect in Project Accept.

**Conclusions:**

In a Southern African setting, HIV incidence estimates and intervention effects can be accurately estimated from cross-sectional surveys using a MAA. The improved accuracy in cross-sectional incidence testing that a MAA provides is a powerful tool for HIV surveillance and program evaluation.

## Introduction

Accurate methods for estimating HIV incidence are needed to monitor the epidemic and evaluate interventions for HIV prevention [1]. In clinical trials, HIV incidence is usually assessed by enrolling HIV-uninfected individuals and following them over time to detect HIV acquisition. An alternate approach is to assess HIV incidence by analyzing specimens from cross-sectional surveys without longitudinal follow-up [2]. This approach may be needed for evaluation of population-level interventions for HIV prevention, particularly when HIV testing is part of a combination prevention strategy [3], [4]. In this report, we describe the development of methods that were used to analyze HIV incidence in a large, Phase III community randomized trial: National Institute of Mental Health (NIMH) Project Accept (HIV Prevention Trials Network 043 [HPTN 043]) [5]. Project Accept is one of the largest randomized, controlled trial performed to date, and is the first randomized controlled trial with a primary study endpoint based solely on cross-sectional estimation of HIV incidence.

Project Accept evaluated the impact of integrated behavioral interventions on HIV incidence in 48 paired communities (34 in Africa, 14 in Thailand) [16]. Control communities received standard, clinic-based, voluntary counseling and testing services; intervention communities received enhanced, community-based voluntary counseling and testing services. After a 3-year intervention period, samples were collected from individuals in the communities (aged 18 to 32 years) in a single cross-sectional survey. When the trial was designed, the study plan was to estimate HIV incidence using the BED capture immunoassay (BED-CEIA, Calypte Biomedical Corporation, Lake Oswego, OR, USA) [6]. That approach was not used because the BED-CEIA was later found to overestimate incidence in many settings [7].

In this report, we describe the laboratory and statistical analysis that was used to identify an alternate testing strategy for HIV incidence estimation in Project Accept. The testing strategies that were evaluated used multiple biomarkers to assess HIV incidence [8]. This approach was based on recent success using a multi-assay algorithm (MAA) to estimate HIV incidence in populations in the United States (clade B settings) [9]–[11]. That MAA combines serologic assays (the BED-CEIA and an antibody avidity assay) with non-serologic biomarkers (CD4 cell count and HIV viral load) to identify individuals who were likely to have been recently infected at the time of sample collection (referred to in this report as MAA positive). In Project Accept, because HIV prevalence in the communities in Thailand was low (<1%, [12]), data from Thailand were not included in the primary endpoint analysis. Therefore, we focused on identifying a MAA that could be used to estimate incidence in the African communities of the trial, using validation samples obtained from seven African cohorts.

Development of methods for cross-sectional HIV incidence estimation is challenging for several reasons. First, an assay or MAA must have a suitable mean window period; this term refers to the average period of time that individuals are identified as positive by a specific assay or MAA. If the window period is too short, fewer individuals will be classified as positive, resulting in higher variance and lower precision of incidence estimates, reducing the power to determine an intervention effect. Conversely, if the window period is too long, the precision of incidence estimates will be reduced because of high bias; furthermore, if too many individuals with long-term infection (e.g, infected >1 year) test positive by an assay or MAA, the incidence estimates will not reflect the current epidemic. Bias is reduced when the probability of being classified as positive approaches zero as the time since infection increases [13], [14]. The performance of serologic assays used for cross-sectional incidence estimation may also be affected by HIV viral load, frequency and duration of antiretroviral treatment (ART), the stage of HIV disease, HIV subtype, and race [15]–[21]. Finally, the performance of assays and MAAs for incidence estimation varies by the stage of the epidemic. For example, a given test method may perform well in an emerging epidemic with high incidence, but may not perform well in waning epidemic where incidence is low and many individuals have advanced HIV disease. In this report, we describe the laboratory and statistical methods used to identify a MAA for incidence analysis in Project Accept.

## Methods

### Samples used for analysis

Samples obtained from seven African cohort studies and clinical trials (Table 1) were used for validation. Samples were selected based on availability of stored plasma, known infecting subtype, known duration of infection (known date of a prior positive and/or negative HIV test), and available CD4 cell count data from the time of sample collection. Of the samples that did not have a known prior HIV negative time point, 99.3% were from individuals who were HIV seropositive for >1 year and 52.7% were known to be from individuals who were HIV seropositive for >2 years. Infection times were either interval-censored (the dates of the first positive and the last negative HIV tests were available) or right-censored (the date of the last negative test was unknown). In the latter case, we assigned the 14th birthday as the date of the last negative test and treated the infection time as interval-censored. Interval-censored infection times were randomly imputed in the simulations. For almost all of the samples, the infection time was generated from the uniform distribution between the last negative and first positive test dates. However, for 147 samples from subjects who had another visit following the current sample date, the infection time was generated from a posterior Weibull density truncated to the interval between the last negative and first positive test dates. We assumed a Weibull distribution on the survival of HIV-infected subjects and a uniform prior distribution of the infection time, and calculated the posterior distribution of the infection time given the date of the last known visit. We used the Weibull survival distribution with the shape parameter 1.856 and rate parameter 4358.5 days. The truncated Weibull density puts more weight on the more recent infection times if the subject is known to have survived for a long time after the first positive test date and so improves the precision of the imputed infection times compared to the uniform distribution. More details on these methods are presented elsewhere [22]. Samples from Botswana, Malawi, South Africa, and Zimbabwe were assumed to come from individuals infected with HIV subtype C [23]. The HIV subtypes of samples from Uganda and Kenya were determined previously [24]–[28].

**Table 1:** Samples used for analysis.

	Gender	Subtype A		Subtype C		Subtype D		All subtypes	
Cohort a	(% female)	# subjects	# samples	# subjects	# samples	# subjects	# samples	# subjects	# samples
CAPRISA	100	0	0	97	552	0	0	97	552
FHI/Uganda	100	46	225	1	3	23	197	70	425
FHI/Zimbabwe	100	0	0	132	339	0	0	132	339
HPTN 039	100	0	0	45	135	0	0	45	135
Partners	64.3	63	155	563	625	18	37	644	817
PEPI	100	0	0	1,663	1,664	0	0	1,663	1,664
Rakai	62.9	254	431	18	37	513	925	785	1,393
Total	84.8	363	811	2,519	3,355	554	1,159	3,436	5,325

a Samples were obtained from the following clinical cohorts (see Methods): CAPRISA: the CAPRISA 004 Study/TRAPS [32]; FHI/Uganda and FHI/Zimbabwe: the FHI360 Hormonal Contraception and HIV (HC-HIV) Trial [24]; HPTN 039: the HIV Prevention Trials Network 039 Trial [33]; Partners: the Partners in Prevention HSV/HIV Transmission Study [34]; PEPI: the Pre-Exposure Prophylaxis in Infants – Malawi Trial [35]; Rakai: the Rakai Health Sciences Program [36].

### Serologic testing

Samples were tested by the BED-CEIA; [6] samples were run in duplicate and the average normalized optical density (OD-n) value was used for analysis. Antibody avidity was measured using a modified version of the Genetic Systems 1/2+O ELISA (BioRad, Hercules, CA) [29]. For this assay, duplicate sample aliquots were diluted 1∶10 and incubated at 4°C for 30 minutes (initial antibody-binding step). Samples were then incubated for 30 minutes at 37°C with or without the chaotropic agent, diethylamine (antibody disassociation step). The avidity index (AI) was calculated as follows: AI  =  [optical density of the diethylamine-treated well]/[optical density of the non-treated well] ×100.

### HIV viral load testing

If a viral load result was not available, viral load testing was performed using the Amplicor HIV-1 Monitor test version 1.5 (Roche Diagnostics, Indianapolis, IN). Viral load testing was only performed for samples that had a BED-CEIA result <1.5 OD-n, an AI <90%, and a CD4 cell count >150 cells/mm^3^.

### Statistical methods

To compare the performance of various testing algorithms, we constructed 403 MAAs that included one or more of the following assays: BED-CEIA, the avidity assay, CD4 cell count, and HIV viral load. The ranges of cutoffs used for each assay were as follows: BED-CEIA: 0.5 to 1.5 OD-n (steps of 0.1 OD-n); the avidity assay: 30% to 90% (steps of 10% AI); CD4 cell count: 150, 200, and 250 cells/mm^3^; all MAAs that included HIV viral load used a cutoff of >400 copies/mL. The MAAs included the BED-CEIA alone, the avidity assay alone, or these two assays in combination with or without inclusion of CD4 cell count; HIV viral load was only included for MAAs that included a CD4 cell count with a cutoff >200 cells/mm^3^.

The mean window period for each MAA was calculated by integrating estimated sensitivity of the MAA, as described [9], with the following caveat: the maximum duration of HIV infection was assumed to be 12 years [22]. The performance of the MAAs for estimating HIV incidence was evaluated in simulated populations with 10% prevalence using three epidemic scenarios (emerging, stable, and waning epidemics). Detailed descriptions of the statistical methods and simulation exercises are described elsewhere [22], The three scenarios were simulated using data from the sample set described in Table 1. For each scenario, data from 200 individuals with different durations of HIV infection were repeatedly sampled. The bias, variance, and root mean square error (RMSE) of the incidence estimate for each of the 403 MAAs was determined for each of the three scenarios. The RMSE measures the overall precision of estimated incidence by combining the bias and variance. The same performance measures were used to evaluate incidence estimates calculated from simulated 6-month and one-year follow-up assessments. In the final evaluation, the data were used to simulate the capacity of selected MAAs to accurately estimate a difference in HIV incidence in the control and intervention communities of Project Accept [22], Finally, the power to detect a 35% decrease in incidence and coverage of confidence intervals for the intervention effect were determined for a stable epidemic setting. The results were compared to simulated intervention effect estimates obtained by 6-month follow-up.

### Human subjects protection

Written informed consent was obtained from study participants and all studies were reviewed and approved by relevant institutional review boards. The study for cross sectional incidence testing on stored study samples was approved by the institutional review board of the Johns Hopkins University. The primary studies for the collection of the samples evaluated were approved by the University of KwaZulu-Natal Biomedical Research Ethics Committee, Medical Research Council of Zimbabwe, Family Health International Protection of Human Subjects Committee, the South African Medicines Control Council, the institutional review board at the University of Malawi, Ethics Committee of the Uganda Virus Research Institute and the Committee for Human Research at Johns Hopkins University and the University of Washington Human Subjects Review Committee.

## Results

### Performance of the BED-CEIA and avidity assay in subtypes A, C, and D

Because HIV subtype can affect performance of the BED-CEIA and avidity assays [18], [30], we first considered the potential impact of HIV subtype on incidence estimation in Project Accept which was conducted in South Africa, Zimbabwe, and Tanzania. Most HIV infections in South Africa and Zimbabwe are subtype C, while Tanzania has three prevalent subtypes: A, C, and D [23]. To evaluate incidence algorithms for use in Project Accept, we obtained validation samples from seven African studies conducted in countries where subtypes A, C, and D are prevalent (Table 1). This sample set included 5,325 samples from 3,436 individuals with known duration of HIV infection (from 1 month to >10 years, see Methods).

**Figure 1:**
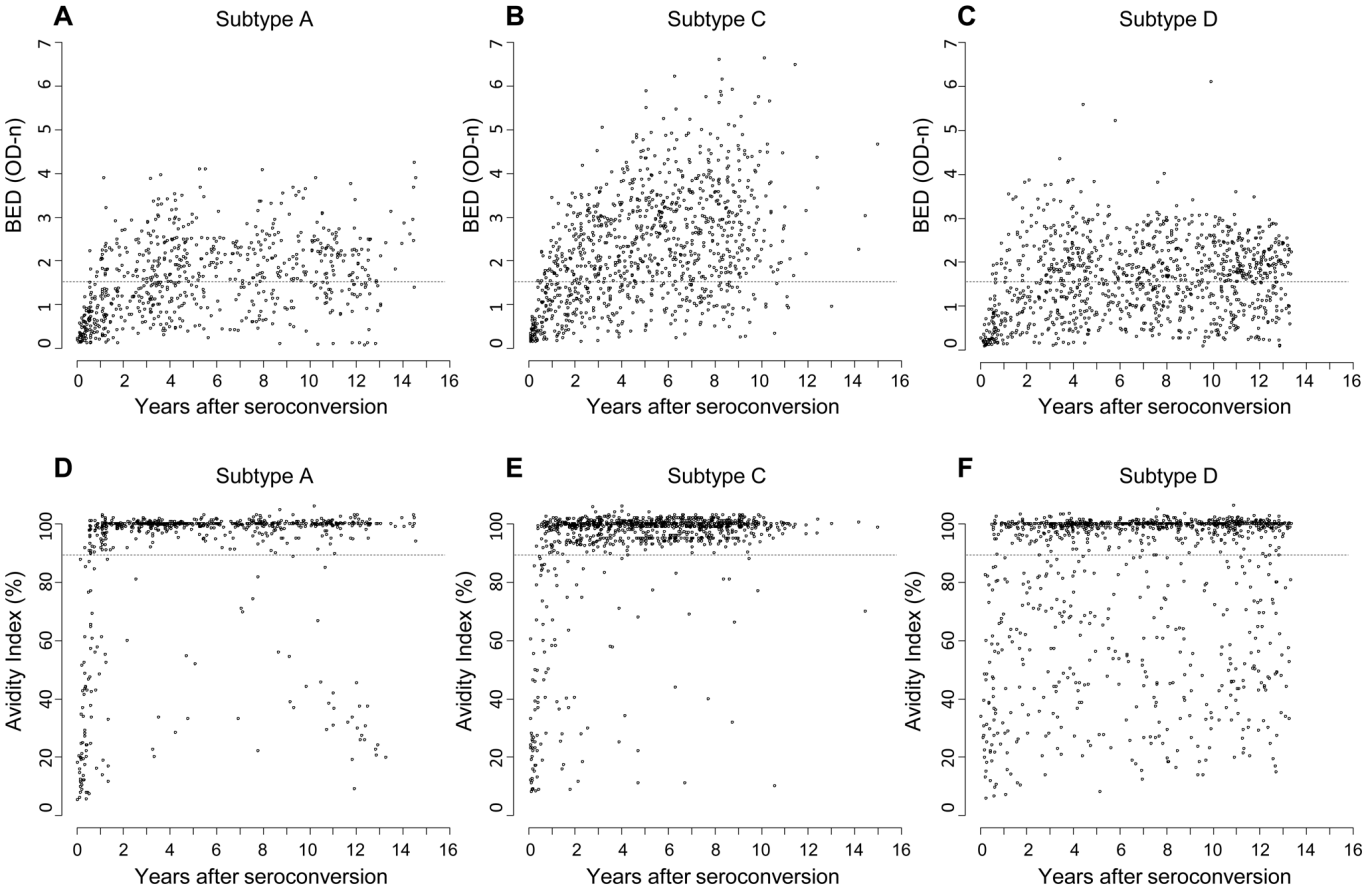
BED-CEIA and avidity assay results for HIV subtypes A, C, and D. Samples from the validation sample set were analyzed using the BED-CEIA (Panels A–C) and the avidity assay (Panels D–F). Results are shown for each assay for subtypes A, C, and D as a function of duration of HIV infection (years after HIV seroconversion). Data are shown for 50 randomly-selected samples for each 6-month interval after seroconversion. The HIV incidence testing algorithms evaluated in this report only included algorithms with BED-CEIA results ≤1.5 OD-n or avidity results ≤90% AI (dashed lines).

First, we evaluated the performance of the BED-CEIA and avidity assay as a function of duration of infection for subtypes A, C, and D (Figure 1). The mean window period for each assay and each subtype were obtained using the standard assay cutoffs (Table 2). For all three subtypes, the mean window period was longer for the BED-CEIA alone than for the avidity assay alone. For both assays, the mean window periods varied considerably among subtypes and were longest for subtype D (>2.5 years for the BED-CEIA, >1.5 years for the avidity assay). We also compared the proportion of individuals infected >2 years who had BED-CEIA results <0.8 OD-n or avidity assay results <40% AI (Table 2). Previous reports indicated that the frequency of subtype D in Tanzania was low [12], [23]; this was confirmed by subtyping a subset of the samples from Project Accept [31]. Because subtype D infections were not likely to have a significant impact on HIV incidence estimates in Project Accept, subsequent analyses did not include subtype D validation samples. After removing the subtype D samples, the validation sample set included 4,166 samples from 2,882 individuals (median age: 27.8 years, interquartile range [IQR]: 24.1 to 32.5 years; 88% women). The performance of the BED-CEIA and the avidity assay was similar for subtypes A and C (Figure 1, Table 2).

**Table 2:** Window periods and classification of individuals with long-standing infection as positive, for the BED-CEIA alone, the avidity assay alone, a two-assay multi-assay algorithm (MAA) and two 4-assay MAAs*.

Assays and assay cutoffs used to identify positive samples	Window periods (years) by HIV subtype (s)				Percentage of samples from individuals infected for >2 years identified as positive			
	A	C	A+C	D	A	C	A+C	D
BED <0.8	2.28	1.45	1.62	2.55	11.90%	7.10%	7.90%	16.00%
AI <40	0.97	0.57	0.65	1.52	5.40%	1.20%	1.90%	8.90%
BED <0.8, AI <70	0.88	0.6	0.67	1.24	2.80%	0.70%	1.10%	6.20%
BED <1.0, AI <80, CD4 >200, VL >400	0.6	0.54	0.56	ND^a^	0.70%	0.70%	0.80%	ND^a^
BED <1.2, AI <90, CD4 >200, VL >400	0.7	0.7	0.7	ND^a^	1.60%	1.40%	1.50%	ND^a^

* Window periods are shown in years for four testing algorithms for subtype A, subtype C, subtypes A and C combined, and subtype D. BED: the BED capture immunoassay (results are expressed as normalized optical density units); Avidity: the avidity assay (results are expressed as a percentage, avidity index); CD4: CD4 cell count (results are expressed as cells/mm^3^); VL: HIV viral load (results are expressed as HIV RNA copies/mL). The lower two rows show results for MAAs (see text); for these MAAs, individuals are classified as MAA positive if they have results for all for assays that are below/above the cutoffs indicated.

a ND: not determined; MAAs that include viral load could not be evaluated for subtype D due to missing viral load data.

### Performance of MAAs for identifying individuals with recent HIV infection

We evaluated the performance of 403 MAAs (see Methods). The mean window period for each MAA is presented in Table S1. Below, we present more detailed information for four of the testing algorithms: the BED-CEIA alone (using the standard assay cutoff of 0.8 OD-n), the avidity assay alone (using the standard assay cutoff of 40% AI)], and two MAAs that include the BED-CEIA, the avidity assay, CD4 cell count and viral load. One of these two MAAs was shown to accurately estimate HIV incidence in subtype B settings (BED-CEIA <1.0 OD-n + AI <80% + CD4 cell count >200 cells/mm^3^ + viral load >400 copies/mL) when testing the samples at the end of follow-up in three clinical studies and comparing the cross-sectional incidence estimates to the incidence observed during study follow-up [9]–[11]. Based on its performance described below, the other MAA was ultimately selected for endpoint analysis in Project Accept (BED-CEIA <1.2 OD-n + AI <90% + CD4 cell count >200 cells/mm^3^ + viral load >400 copies/mL) [22].

**Figure 2:**
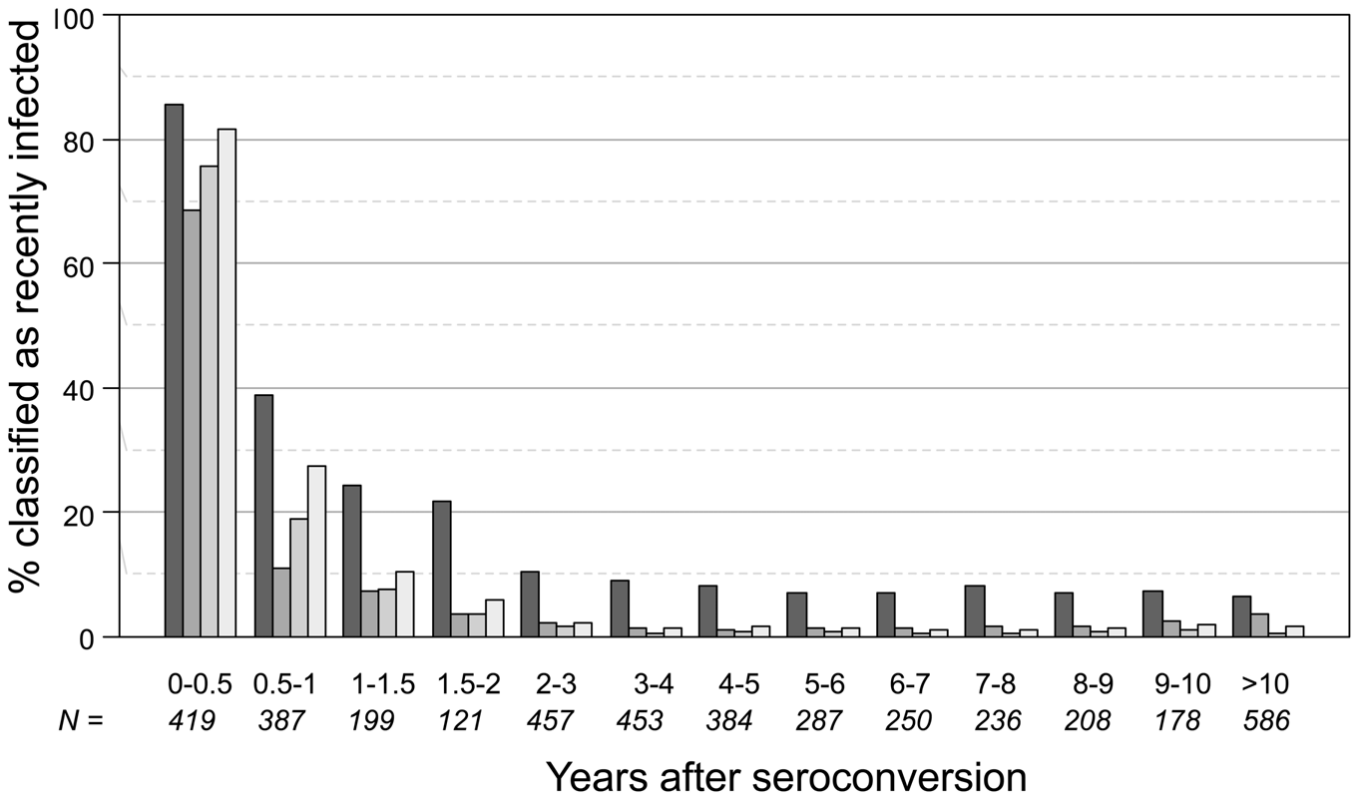
Proportion of samples classified as positive using the BED-CEIA alone, the avidity assay alone, and two MAAs. Subtype A and C samples were analyzed using the BED-CEIA alone (using the standard assay cutoff of 0.8 OD-n, black bars), the avidity assay alone (using the standard assay cutoff of 40% AI, dark grey bars), and two MAAs that included multiple biomarkers, (BED-CEIA <1.0 OD-n + AI <80% + CD4 cell count >200 cells/mm^3^ + viral load >400 copies/mL, medium grey bars; BED-CEIA <1.2 OD-n + AI <90% + CD4 cell count >200 cells/mm^3^ + viral load >400 copies/mL, light grey bars). For each test method, the percentage of samples classified as positive was determined as a function of the duration of HIV infection (years after HIV seroconversion). N indicates the number of samples analyzed for each time period (e.g., 0–0.5 years after seroconversion).

The proportion of samples positive for each of the four testing algorithms was determined as a function of duration of infection (Figure 2). Consistent with results shown in Figure 1, a high proportion of individuals infected >2 years were positive by the BED-CEIA alone (7.9%). The other three testing approaches identified a lower proportion of these long-term infections as positive (1.9% for the avidity assay, 0.8% and 1.5% for the 4-assay MAAs). For individuals infected <6 months, 68.5% were positive using the avidity assay alone, 75.6% were positive using the MAA that was previously optimized for incidence estimation in clade B epidemics, and 81.5% were positive using the MAA that was ultimately selected for analysis of the Project Accept endpoint. The MAA that was ultimately selected for use in Project Accept identified 27.5% of those infected 0.5–1 year and 8.8% of those infected 1–2 years as positive.

### Performance of MAAs for estimating HIV incidence in different epidemic scenarios

**Figure 3:**
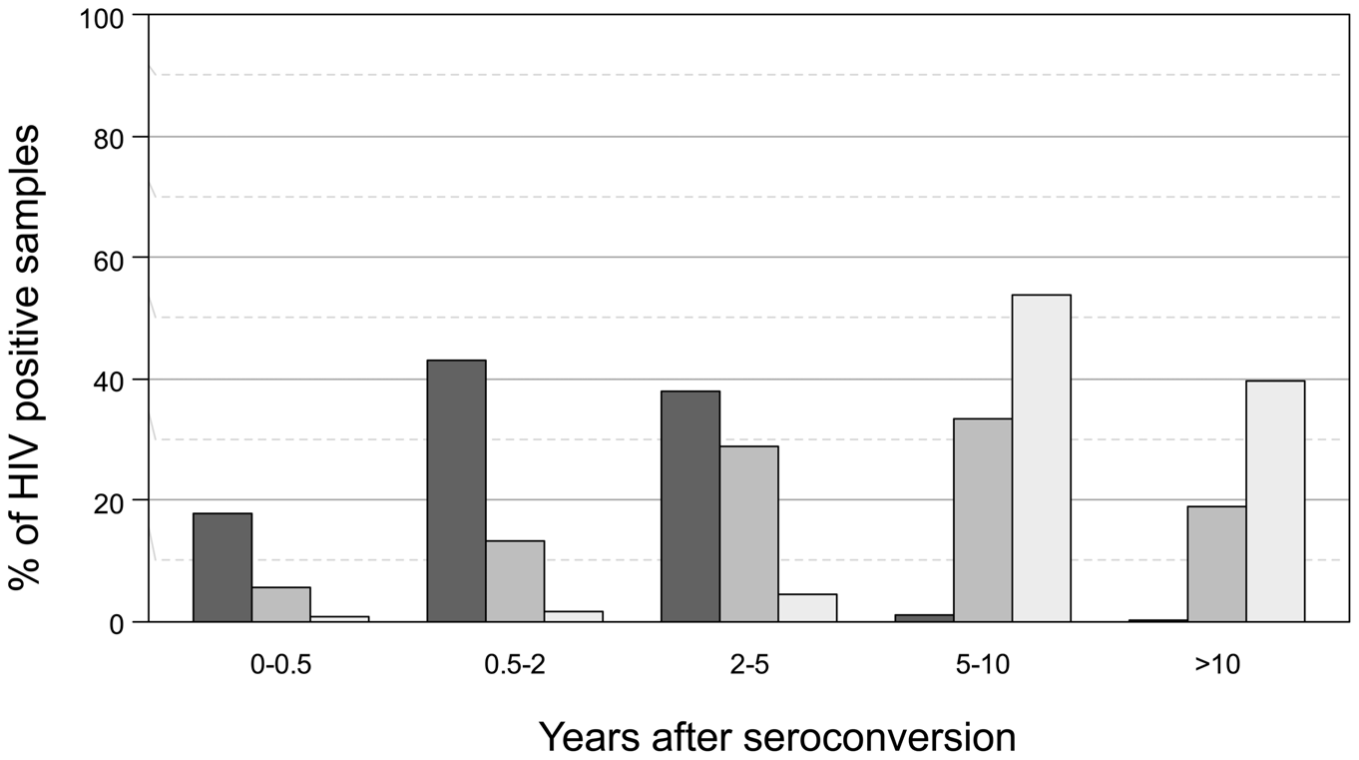
Simulated epidemic scenarios. HIV incidence testing algorithms were assessed using three simulated epidemic scenarios: an emerging epidemic (black bars), a stable epidemic (dark grey bars), and a waning epidemic (light grey bars). The plot shows the percentage of HIV-positive samples included in each scenario for different time periods (years after HIV seroconversion).

The overall performance of incidence assays and MAAs is affected by the distribution of infection times in a population. Because the stage of the HIV epidemic in the Project Accept communities was not known, we evaluated the performance of the 403 MAAs for estimating HIV incidence in three simulated epidemic scenarios: emerging, stable, and waning epidemics. These scenarios were constructed by randomly selecting samples from the validation data set so that the distribution of durations of infection corresponded to the desired scenario (see Methods, Figure 3). In the stable epidemic, 5.5% of individuals were infected <0.5 years, 13.3% were infected 0.5–2 years, 29.0% were infected 2–5 years, 33.4% were infected 5–10 years, and 17.8% were infected >10 years. For the emerging epidemic, these percentages were 17.7%, 42.9%, 38.4%, and 1.0%, respectively; for the waning epidemic, these percentages were 0.7%, 1.6%, 44.0%, and 53.7%, respectively.

The bias, variance, and RMSE were calculated for each MAA in the three epidemic scenarios. Results obtained using the BED-CEIA alone, the avidity assay alone, the best performing 2-assay MAA (BED-CEIA <0.8 OD-n + AI <70%) and the two 4-assay MAAs described above are shown in Table 3; results for all 403 MAAs are shown in Supplemental Table 1. The bias reflects the difference between the estimated incidence and the true incidence. Among the 403 MAAs, the bias ranged from −6.1% to −63.3% in the emerging epidemic scenario, from −9.2% to −26.6% in the stable epidemic scenario, and from 40.6% to 346.7% in the waning epidemic scenario. In the stable and emerging epidemic scenarios, the bias was lower for MAAs that used serologic assays in combination with CD4 cell count and viral load (Table S1). The RMSE ranged from 0.20 to 1.01 in the emerging epidemic scenario, from 0.28 to 0.55 in the stable epidemic scenario, and from 0.65 to 1.50 in the waning epidemic scenario. The best performing two assay MAA was ranked 186 out of the 403 algorithms tested. Though this MAA was comparable to the best 3- and 4-assay MAAs in the waning epidemic scenario, it performed poorly in stable and emerging scenarios, In the emerging and waning epidemic scenarios, the lowest precision values (highest RMSEs) were obtained using the BED-CEIA alone or the avidity assay alone.

**Table 3:** Accuracy of incidence estimates obtained using the BED-CEIA alone, the avidity assay alone, a two-assay multi assay algorithm (MAA), and two four-assay MAAs in three epidemic scenarios*.

		Epidemic scenario									
		Stable epidemic			Emerging epidemic			Waning epidemic			
	Window Period	(annual incidence 1.29%)			(annual incidence 4.18%)			(annual incidence 0.16%)			
Algorithm	(years)	Rank	Rel. bias	RMSE	Rank	Rel. bias	RMSE		Rank	Rel. bias	RMSE
6-month follow-up	–	–	−7.9%	0.32	–	−5.7%	0.17		–	18.6%	0.51
BED <0.8	1.63	95	−23.2%	0.32	396	−49.9%	0.70		396	221.4%	1.19
AI <40	0.67	339	−24.5%	0.42	390	−37.0%	0.49		390	149.7%	1.06
BED <0.8, AI <70	0.67	257	−20.2%	0.38	309	−25.4%	0.33		29	50.0%	0.71
BED <1.0, AI <80, CD4>200, VL>400	0.56	125	−9.9%	0.33	7	−13.1%	0.21		20	49.0%	0.71
BED <1.2, AI <90, CD4>200, VL>400	0.71	23	−11.4%	0.29	91	−17.6%	0.24		78	64.2%	0.75

* MAA: multi-assay algorithm; BED-CEIA: BED capture immunoassay (results expressed as normalized optical density units); AI: avidity index (results expressed as a percentage); CD4: CD4 cell count (results expressed as cells/mm^3^); VL: viral load (results expressed as HIV RNA copies/mL); yrs: years; Rel. bias: relative bias; RMSE: root mean square error. The lower two rows show results for MAAs (see text); for these MAAs, individuals are classified as MAA positive if they have results for all for assays that are below/above the cutoffs indicated.

The relative bias (in % of true incidence over 12 months) and precision of incidence estimates (expressed as the root mean square error for log incidence, RMSE) are shown for a 6-month cohort follow-up estimator and four cross-sectional testing algorithms in three different epidemic scenarios. The ranks show the relative ranking of each algorithm among the 403 evaluated algorithms according to precision of incidence estimation (RMSE).

Finally, we compared the capacity of the four testing algorithms to accurately detect a 35% difference in HIV incidence in the control and intervention communities of Project Accept (Table 4) [22]. The intervention effect was accurately estimated by both of the 4-assay MAAs, but was underestimated using BED-CEIA alone or the avidity assay alone. The percentage of 95% confidence intervals that covered the true intervention effect was >93% for both 4-assay MAAs, but was unacceptably low for the BED-CEIA alone or the avidity assay alone. The MAA that was ultimately selected for primary endpoint analysis in Project Accept (BED-CEIA <1.2 OD-n + avidity index <90% + CD4 cell count >200 cells/mm^3^ + viral load >400 copies/mL) provided the largest power for detecting a 35% reduction in HIV incidence and had minimal bias in estimating incidence across differing epidemic scenarios. This MAA had better precision, power, and negligible bias compared to a simulated 6-month follow-up study using the same validation sample set. In a separate simulation, we showed that both of the 4-assay MAAs maintained the required probability of type I error, provided that the scenarios in the paired communities were the same.

**Table 4:** Capacity to estimate and detect a 35% reduction in HIV incidence in the Southern African communities of Project Accept using the BED-CEIA alone, the avidity assay alone, and two multi-assay algorithms (MAAs)*.

Algorithm	Estimated intervention effect (RR)	Std. error of log estimated RR	Power	Coverage of 95% confidence intervals for RR
6-month follow-up	0.631	0.182	70.4%	94.7
BED <0.8	0.763	0.109	68.4%	57.3
AI <40	0.705	0.165	56.5%	88.8
BED <1.0, AI <80, CD4 >200, VL >400	0.653	0.169	69.7%	95.6
BED <1.2, AI <90, CD4 >200, VL >400	0.663	0.157	75.5%	93.1

* BED-CEIA: BED capture immunoassay (results expressed as normalized optical density units); AI: avidity assay (results expressed as a percentage, avidity index); CD4: CD4 cell count (results expressed as cells/mm^3^); VL: viral load (results expressed as HIV RNA copies/mL); Std: standard; RR: relative risk.

The table shows the mean estimated intervention effect, empirical standard error of log estimated intervention effect, the power to detect the 35% difference in incidence, and the coverage of the 95% confidence intervals obtained by a simulation study under the stable epidemic scenario. The lower two rows show results for MAAs (see text); for these MAAs, individuals are classified as MAA positive if they have results for all for assays that are below/above the cutoffs indicated.

## Discussion

We evaluated the performance of incidence assays and MAAs using data from a large set of validation samples from Africa. These samples were from individuals with a broad range of infection times who had CD4 cell count data available from the time of sample collection. We found that testing algorithms that included multiple assays were superior to single serologic assays; the incidence estimates obtained using multiple assays had lower bias and better precision. We used simulation exercises to demonstrate that the 4-assay MAA that was selected for use in Project Accept provided a more precise estimate of the ratio between incidence in the intervention and control communities than would have been obtained by following a cohort for seroconversion over a 6-month period.

Our findings demonstrate the importance of including samples from very long-term infections when validating methods for cross-sectional HIV incidence estimation. Using a sample set that included individuals infected >10 years, the mean window period for the BED-CEIA was 1.63 years, which is approximately three times longer than the mean window period previously reported for this assay [18]. We found that the BED-CEIA and avidity assay frequently identified individuals infected >2 years as positive. This effect was most pronounced for subtype D. Previous studies have shown that misclassification of subtype D samples using these assays reflects differences in the serologic response to subtype D infection compared to subtype A infection; differences in the sequences of subtype D viruses in the region corresponding to the BED-CEIA target antigen also negatively affect assay performance [30]. We recommend against using the BED-CEIA or Bio-Rad avidity assays for cross-sectional incidence estimation in populations that include a substantial proportion of subtype D-infected individuals. Additional studies should be performed using different test methods [e.g., different assay (s) and/or different cutoff (s)] to identify an effective method for cross-sectional incidence estimation in subtype D endemic areas. Fortunately, the prevalence of subtype D was low in the Tanzanian communities in Project Accept [31]. Therefore, we felt it was reasonable to use a MAA that was optimized for subtypes A and C for estimating incidence in the four African sites in Project Accept.

In this report, we used a novel approach to compare the performance of a large set of MAAs that employed different combinations of assays and assay cutoffs in three different simulated epidemic scenarios. The MAAs that included both serologic assays and non-serologic biomarkers (CD4 count and viral load) had lower bias and variance for estimating incidence than algorithms based on a single assay. The MAA that provided the greatest power to detect a reduction in incidence in Project Accept used relatively high cutoffs for both the BED-CEIA (<1.2 OD-n) and the avidity assay (<90% AI), which increased the identification of individuals infected within 1 year. Use of these higher cutoffs for increased the frequency of misclassification, though it was still <1.5% for individuals infected >2 years.

HIV viral load was included in 77 of the 403 MAAs evaluated in this study. This biomarker identifies both elite suppressors and individuals who are virally suppressed from ART. It is important to identify both of these groups, since viral suppression is associated with down-regulation of the humoral immune response to HIV infection and with increased rates of false-recent misclassification using serologic HIV incidence assays [2]. ART also serves as an independent surrogate for non-recent HIV infection, since individuals with recent HIV infection are not likely to be identified or to access ART in many settings. We recognize that the 4-assay MAA selected for endpoint analysis in Project Accept may misclassify some individuals on ART who are not virally suppressed (e.g., those with low viral loads that are >400 copies/ml). This was addressed in Project Accept using a two-step approach: first, the MAA was used to identify samples from individuals who were likely to have had recent infection; second, these samples were tested for the presence of antiretroviral drugs [31]. When using antiretroviral drug testing in an HIV incidence assessment, it is important to consider the test results in the context of antiretroviral drug regimens that were used in the communities at the time the incidence survey was conducted; this should take into account use of antiretroviral drugs for HIV prevention (e.g., for prevention of mother-to-child transmission, where those receiving prophylaxis may have been recently infected).

The novel approach that was used to select an optimal testing algorithm for HIV incidence assessment in Project Accept is of general relevance to HIV prevention trials. We demonstrate that a large validation data set from individuals with known duration of infection can be used to assess the performance of various testing algorithms in terms of estimating incidence, providing estimates of bias and precision, and comparing the cross-sectional estimates to cohort-based estimates. We also used an empirical approach to determine assay cutoffs that optimized the precision of cross-sectional incidence estimates using MAAs. The methods described in this report could be used for cross-sectional incidence assessment in non-subtype D epidemics of Southern Africa for HIV prevention studies, surveillance, and other purposes.

## Supporting Information

Table S1 Window periods, bias, root square mean error (RMSE) for stable, emerging and waning epidemics for 403 multi-assay algorithms (MAAs). *
*BED: BED capture immunoassay (results expressed as normalized optical density units); AI: avidity assay (results expressed as a percentage, avidity index); CD4: CD4 cell count (results expressed as cells/mm^3^); VL: viral load (results expressed as HIV RNA copies/mL); yrs: years;
(XLS)
